# Simultaneous Reality Filtering and Encoding of Thoughts: The Substrate for Distinguishing between Memories of Real Events and Imaginations?

**DOI:** 10.3389/fnbeh.2017.00216

**Published:** 2017-11-01

**Authors:** Raphaël Thézé, Aurélie L. Manuel, Louis Nahum, Adrian G. Guggisberg, Armin Schnider

**Affiliations:** Laboratory of Cognitive Neurorehabilitation, Division of Neurorehabilitation, Department of Clinical Neurosciences, University Hospital and University of Geneva, Geneva, Switzerland

**Keywords:** source memory, evoked potentials, connectivity, theta band, orbitofrontal cortex, reality monitoring, orbitofrontal reality filtering, encoding

## Abstract

Any thought, whether it refers to the present moment or reflects an imagination, is again encoded as a new memory trace. Orbitofrontal reality filtering (ORFi) denotes an on-line mechanism which verifies whether upcoming thoughts relate to ongoing reality or not. Its failure induces reality confusion with confabulations and disorientation. If the result of this process were simultaneously encoded, it would easily explain later distinction between memories relating to a past reality and memories relating to imagination, a faculty called reality monitoring. How the brain makes this distinction is unknown but much research suggests that it depends on processes active when information is encoded. Here we explored the precise timing between ORFi and encoding as well as interactions between the involved brain structures. We used high-density evoked potentials and two runs of a continuous recognition task (CRT) combining the challenges of ORFi and encoding. ORFi was measured by the ability to realize that stimuli appearing in the second run had not appeared in this run yet. Encoding was measured with immediately repeated stimuli, which has been previously shown to induce a signal emanating from the medial temporal lobe (MTL), which has a protective effect on the memory trace. We found that encoding, as measured with this task, sets in at about 210 ms after stimulus presentation, 35 ms before ORFi. Both processes end at about 330 ms. Both were characterized by increased coherence in the theta band in the MTL during encoding and in the orbitofrontal cortex (OFC) during ORFi. The study suggests a complex interaction between OFC and MTL allowing for thoughts to be re-encoded while they undergo ORFi. The combined influence of these two processes at 200–300 ms may leave a memory trace that allows for later effortless reality monitoring in most everyday situations.

## Introduction

Based on the observation of patients who suffer from confabulations and disorientation, we have described a mechanism which appears to filter upcoming thoughts and memories according to their relation with current, ongoing reality (Schnider, 2003, 2008). We call this mechanism “Orbitofrontal Reality Filtering” (ORFi; Schnider, 2013). It depends on the orbitofrontal cortex (OFC), as indicated by lesion analysis of patients (Schnider et al., 1996c; Schnider and Ptak, 1999; Schnider, 2008) and functional imaging in healthy subjects (Schnider et al., 2000; Treyer et al., 2003, 2006). The experimental task measuring this capacity consists of repeated runs of a continuous recognition task (CRT) composed of the same picture set but presented in a different order in each run. Subjects were asked to indicate picture repetitions within the ongoing run only. Reality-confusing patients strikingly increased their false positive rate from the second run on, believing that they had already seen the pictures within the ongoing run (the “current reality”; Schnider et al., 1996a; Schnider and Ptak, 1999; Nahum et al., 2012). In healthy subjects, correct response to such stimuli evoked a frontal positivity at about 200–300 ms (Schnider et al., 2002; Wahlen et al., 2011; Bouzerda-Wahlen et al., 2015), which emanated from the posterior medial OFC (Schnider et al., 2000; Bouzerda-Wahlen et al., 2015). Thus, this frontal positivity seems to signal that an upcoming thought (memory) does not pertain to the ongoing reality. In the present study, we used this frontal potential as a marker for ORFi.

ORFi determines whether an upcoming thought refers to present reality and to one’s current role. This is different from the ability to determine whether one actually experienced an event in the past or only thought about it (imagination), a capacity called reality monitoring (Johnson and Raye, 1981). Its mechanism is unclear. While it is mostly characterized as a retrospective monitoring function (Johnson and Raye, 1981; Johnson et al., 1993; Mitchell and Johnson, 2009), it is unclear what criteria, other than plausibility, the brain would apply to verify such information at retrieval, considering that, at this stage, there is only the memory trace. In most daily situations, elaborate monitoring is not necessary; we easily realize whether we have actually been in a certain place the day before or only thought about it. An alternative hypothesis would be that the substrate of reality monitoring is established as we experience a situation: thoughts that relate to reality would be stored in a different format than thoughts reflecting imaginations. Indeed, multiple studies pointed to the importance of activity at encoding for later reality monitoring (Davachi et al., 2003; Gonsalves et al., 2004; Kensinger and Schacter, 2005; Sugimori et al., 2014). Specifically, we suggest that reality monitoring would be easy to explain if evoked thoughts were encoded while they underwent ORFi at 200–300 ms.

Unfortunately, there is no generally recognized evoked potential marker of encoding, especially one that could be integrated in a CRT. New stimuli differ from repeated ones essentially by relatively late (>400 ms) posterior amplitude variations, which might reflect recognition or encoding (Schnider et al., 2002; Rugg and Curran, 2007). However, we have observed that immediately repeated stimuli in a CRT evoked a frontal positivity at about 200–300 ms, which emanated from the medial temporal lobe (MTL), as suggested by source estimation (James et al., 2009) and subsequently confirmed with depth electrode measurements in epileptic patients (Nahum et al., 2011). This potential was associated with increased functional connectivity (FC) of the MTL with the rest of the brain (Thézé et al., 2016). Most importantly, this potential has a protective effect on the memory trace—subjects having strong MTL connectivity to the rest of the brain in this period had better overall recognition after 30 min (Thézé et al., 2016). Conversely, amnesic subjects weakly express or lack this potential (Nahum et al., 2015). In the present study, we used this frontal potential as a surrogate marker of memory encoding.

In the present study, we tested the hypothesis that encoding of upcoming thoughts is initiated either slightly before or simultaneously with the verification of their relation with ongoing reality, thus forming a plausible basis for later reality monitoring. Therefore, we devised an experiment combining the challenges of encoding and ORFi within a single CRT. The goals of the study were: (1) to compare the precise timing between ORFi and encoding of thoughts (memories), as reflected in the frontal potentials representative of the two processes; and (2) to test for interactions between the posterior OFC, the MTL and the neocortex using FC analyses.

## Materials and Methods

### Participants

Twenty-one healthy subjects with no history of neurological or psychiatric illness gave written informed consent and were paid to take part in the study. Three subjects were subsequently excluded due to a high level of noise in the electroencephalography (EEG) data leaving 18 subjects to further analysis (9 women; age 24.8 ± 11.9 years, 20–34). Four of them were left-handed according to the Edinburgh Handedness Inventory (Oldfield, 1971). This study was approved by the ethics committee of Geneva, the Commission Cantonal d’Ethique pour la Recherche (CCER). All subjects gave written informed consent in accordance with the Declaration of Helsinki.

### Paradigm

Subjects performed a CRT combining the encoding task described by James et al. (2009) and the reality filtering task described by Schnider et al. (2002).

**Figure 1:**
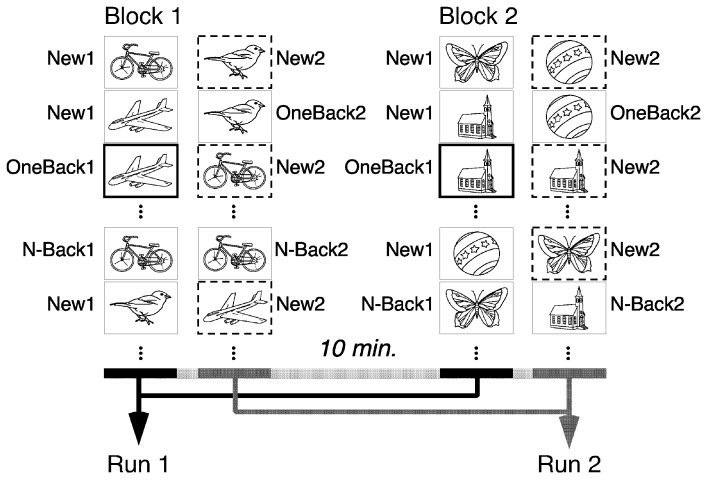
Design of the task. Both runs are composed of the same set of pictures, re-arranged in a different order. Subjects have to indicate repetitions within, and only within the ongoing run. There were six types of stimuli: New1,2 are first-appearances within run1 or 2. OneBack1,2 are immediate repetition within run1,2. N-Back1,2 are repetitions after 8–12 intervening items within run1,2. Items in bold were the critical markers for encoding (OneBack1). Items with dashed border are the critical stimuli for Orbitofrontal reality filtering (ORFi; New2). Data of the two blocks were pooled for the analysis.

The task consisted of two runs of a CRT, both composed of the same set of 60 meaningful line drawings from Snodgrass and Vanderwart (1980), but arranged in different order in the two runs (Figure 1). In both runs, each picture was repeated either immediately after its initial appearance (OneBack; 30 per run) or after 8–12 intervening stimuli (N-Back; 30 per run). In both runs, subjects had to indicate by button press with their right middle or index finger whether they had already seen the presented picture “within, and only within” the currently ongoing run or not. The two runs were separated by 1 min, the time needed to start presentation of run 2.

In the first run, all stimuli are initially new. Thus, this run measures learning and recognition of new information, associated with activation of the MTL (Schnider et al., 2000). In the present study, the stimuli of main interest were the immediate repetitions of this first run (One-Back1), whose evoked potential response appears to reflect a MTL-mediated encoding process (James et al., 2009; Nahum et al., 2011; Thézé et al., 2016). Processing of OneBack1 (in comparison with initial presentations and delayed repetitions in the first run), was, therefore, the surrogate marker for Encoding in this study.

In the second run, all stimuli are already familiar. Performing this second run thus requires the ability to sense when a stimulus occurring for the first time in this run (new within this second run; New2) is not a repetition, a capacity depending on activation of the OFC (Schnider et al., 2000). Processing of New2 stimuli (in comparison with first appearances in the first run and delayed repetitions in both runs) was, therefore, the surrogate marker for ORFi in this study.

In order to obtain enough triggers for EEG analysis and prevent fatigue of the participants, subjects made a second block with two runs after a 10-min break. This block was composed of a completely new set of pictures. Task design was identical to the first block.

Data of the two blocks were pooled for analysis. Thus, the pooled first runs contained 120 initial presentations (New1), 60 immediate repetitions (OneBack1) and 60 delayed repetitions (N-Back1). The pooled second runs contained 120 initial presentations (New2), 60 immediate repetitions (OneBack2) and 60 delayed repetitions (N-Back2), yielding a total of 480 stimulus presentations.

Stimuli were presented on a 17-inch monitor positioned at eye-level, at the size of 8° of visual angle for 1000 ms. Interstimulus interval was 2000 ms, filled with a fixation cross.

### Behavioral Data Analyses

The percentage of correct responses (%Hits) and reaction times (RT) were analyzed with 2 × 3 repeated measures ANOVAs (rmANOVAs) using Run (Run1, Run2) and Stimulus (New, OneBack, N-Back) as within-subject factors. In case of significant interaction, *post hoc* paired *t*-tests were performed, Bonferroni corrected by the number of factors. Effect sizes are reported as partial eta square ($\eta_{\text{p}}^{\text{2}}$), which is the proportion of the effect with the error variance attributable to this effect.

### Data Acquisition

Recordings were made using PyCorder software with a 156-channel Brain Products EEG machine equipped with BrainVision actiCHamp amplifier and actiCAP active electrodes (Brain Products GmbH, Germany). Data were sampled at 500 Hz. The impedance at each electrode was kept under 20 kΩ. The recordings were made in a soundproof Faraday cage, either in the morning or the afternoon.

### ERP Analysis

Pre-processing and inverse solutions were done with the Cartool software developed by Denis Brunet^1^. The trials of each condition were aligned to the onset of visual stimulus presentation and epochs from 100 ms pre-stimulus to 600 ms post-stimulus onset were extracted. Data were band-pass filtered (3–45 Hz), baseline corrected using the 100 ms pre-stimulus period and recalculated against the average reference. In addition to a criterion of ±100 μV, artifacts such as eye blinks, eye movements, muscular contractions, or electrodes artifacts were excluded by visual inspection. Bad channels containing recurrent artifacts over prolonged periods were interpolated from neighboring electrodes using a 3D spline interpolation (<5% interpolated electrodes; Perrin et al., 1987). Artifact-free EEG epochs were averaged as a function of Stimulus (New, OneBack, N-Back) and Run (Run1 and Run2).

#### Global Waveform Analysis

Electrode- and time-wise ANOVAs were conducted for each of the 156 electrodes for the Encoding and ORFi conditions separately. For the Encoding condition, a one-way rmANOVA including New1, OneBack1 and N-Back1 stimuli was performed. For the ORFi condition, a 2 × 2 repeated measures ANOVA with factors Run (1,2) and Stimulus type (New, N-Back1) was performed. This analysis was done with the Statistical Toolbox for Electrical Neuroimaging (STEN) developed by Jean-François Knebel^2^. To account for temporal autocorrelation, only periods that remained significant (*p* < 0.01) for 10 contiguous data sampling points (≥20 ms) were considered reliable (Guthrie and Buchwald, 1991; Toepel et al., 2014; Manuel and Schnider, 2016). This analysis served to define periods of interest in data-driven way, with no* a priori* choice of periods of interest. To assess the direction of effects and the presence of the specific encoding and ORFi markers, follow-up analyses were performed in a frontal cluster.

#### Frontal Cluster Waveform Analysis

The main waveform analysis concerned the frontal potentials associated with ORFi and Encoding. Since the electrophysiological correlate of the ORFi potential (response to New2 items) and encoding potential (response to OneBack1 items) are expressed in the frontal region (Schnider et al., 2002; James et al., 2009), we analyzed frontal amplitude modulations averaged over eight frontal electrodes in each subject and for each condition over the 600 ms post-stimulus onset period, as previously done by Bouzerda-Wahlen et al. (2015). The cluster of eight electrodes corresponded to the Fz, AFz, F2, AF4, AFF1h, AFF2h, AFp1 and AFp2 electrodes position of the 160Ch Standard Electrode Layout for actiCHamp based on the 10/20 system. The analyses were performed in Matlab (The MathWorks Inc., Natick, MA, USA).

For each time period with significant effects in the global waveform analysis, amplitude differences between stimuli relevant to Encoding and ORFi were separately sought using repeated-measure ANOVAs. In the case of significant interactions, paired *t*-tests were performed.

The presence of a specific electrocortical response reflecting Encoding was verified with a one-way rmANOVA including New1, OneBack1 and N-Back1 stimuli, with special interest in the response to OneBack1. The presence of a specific response reflecting ORFi was verified with a 2 × 2 repeated measures ANOVA with factors Run (1,2) and Stimulus type (New, N-Back) with special interest in the response to New2. After confirming the presence of the potentials specific for Encoding (response to OneBack1) and for ORFi (response to New2) we compared their onsets (beginning of the positive deflection) and maximum amplitudes. Onset and peak amplitudes were measured separately at single subject level for both the encoding and the ORFi potentials and were then statistically compared using paired *t*-tests.

#### Source Analysis

To estimate the brain areas activated in Encoding and in ORFi, a distributed linear inverse solution based on Local Auto-Regressive Average (LAURA) was applied over periods with significant effects in the waveform analysis (Grave de Peralta Menendez et al., 2001, 2004; Michel and Murray, 2012). The solution space is based on a 3D realistic head model comprising 4146 nodes distributed within the gray matter of the average brain provided by the Montreal Neurological Institute (MNI). LAURA provides a current density value (μA/mm^3^) for each node. For each period of interest, local electrical current densities were statistically compared with time-point wise paired *t*-tests using Cartool. To correct for temporal autocorrelation and multiple testing, only periods with *p* < 0.01 for at least 20 ms were retained (Guthrie and Buchwald, 1991; Toepel et al., 2014; Manuel and Schnider, 2016).

### Functional Connectivity

We explored network dynamics by means of a FC analysis. This global analysis has the advantage of being independent of choice of time, electrodes, frequencies and regions of interest (ROI). It was performed in Matlab using the Functional Connectivity Mapping (FCM) toolbox (Guggisberg et al., 2011) in NUTMEG (Dalal et al., 2011)^3^.

We used an event-related approach allowing us to assess the temporal evolution of neural interactions (Andrew and Pfurtscheller, 1996), as previously used by Thézé et al. (2016). First neural oscillations at each trial were reconstructed in the inverse solution space. To this end, we computed a lead potential with 10 mm grid spacing using a *spherical head model with anatomical constraints* (SMAC; Spinelli et al., 2000) derived from the segmented MNI MRI template brain. A scalar minimum variance beamformer was used to reconstruct gray matter oscillations from the surface EEG recordings (Sekihara et al., 2004). EEG was bandpass-filtered between 3 and 45 Hz, which optimized the beamformers for those frequencies, and projected to gray matter voxels with an adaptive spatial filter calculated for each subject from all artifact-free epochs of a given condition in the bandwidth of interest, using the entire epoch duration (Guggisberg et al., 2011). EEG epochs were Fourier transformed using a sliding Hanning window of 300 ms width shifted in time steps of 50 ms. The Event-Related Coherence (ERCoh) was then computed for each stimulus type separately as the magnitude squared coherence between all gray matter voxel pairs, at each time window and through four defined frequency bands (*theta* 3–7.5 Hz, *alpha* 7.5–12 Hz, *beta* 12.5–30 Hz and *gamma* 30–40 Hz). Global FC was calculated as the average ERCoh of a given voxel with all other voxels of the brain. Baseline ERCoh (−400 to 0 ms before stimulus onset) was subtracted from all time windows. This also removed confounds due to volume conduction.

For each condition, ERCoh differences were tested against the null-hypothesis of zero change with statistical non-parametric mapping (SnPM) at each voxel. Then, we performed a paired comparison between stimuli New1, OneBack1 and N-Back1 to investigate connectivity during Encoding and with stimuli New1, New2, N-Back1 and N-Back2 to investigate connectivity during ORFi. Cluster corrections for multiple testing were obtained with group permutations and by defining a voxel cluster-size threshold such that clusters spatially larger than this threshold are significant at *p* < 0.01 (Singh et al., 2003).

To further investigate neural interactions during Encoding and ORFi, we additionally performed a seed analysis of coherence. Based on results from the global connectivity analysis, we defined the right hippocampus, as part of the MTL, and the gyrus rectus, as part of the OFC, as anatomical ROIs with the Automated Anatomical Labeling (AAL) atlas and then computed the global mean ERCoh change across its voxels. Finally, the ROIs were defined as seed area and for each condition ERCoh differences were tested as before with SnPM at each voxel.

## Results

### Behavioral Analysis

Table 1 summarizes the behavioral results. Repeated measures ANOVA revealed that immediate repetitions (OneBack) were processed more accurately (main effect of Stimulus, *F*_(2,34)_ = 4.82, *p* = 0.014, $\eta_{\text{p}}^{\text{2}}$ = 0.22) and faster (main effect of Stimulus, *F*_(2,34)_ = 44.45, *p* < 0.01, $\eta_{\text{p}}^{\text{2}}$ = 0.72) than New and N-Back stimuli. Additionally, there was a significant interaction between Stimulus and Run for RT (*F*_(2,34)_ = 3.57, *p* = 0.04, $\eta_{\text{p}}^{\text{2}}$ = 0.17). *Post hoc* Bonferroni corrected pairwise comparisons revealed that OneBack stimuli were processed faster than New and N-Back stimuli in both runs (*p* < 0.01). The interaction was due to faster RTs in response to OneBack in the second run.

**Table 1:** Behavioral results.

	Stimuli	Hits (%)	SD (±)	RT (ms)	SD (±)
Run 1	New	95.1	2.62	787.6	109.0
	OneBack	97.8	2.56	671.5	83.44
	N-Back	93.5	9.09	804.9	100.58
Run 2	New	94.7	3.67	807.9	128.65
	OneBack	98.6	2.37	652.0	89.37
	N-Back	93.1	9.05	820.2	112.43

*Average correct answers (Hits) in percent and Reaction Time (RT) in ms of all subjects with standard deviation (SD) for each stimulus (New, OneBack and N-Back) during run 1 and 2*.

### Global Waveform Analysis

**Figure 2:**
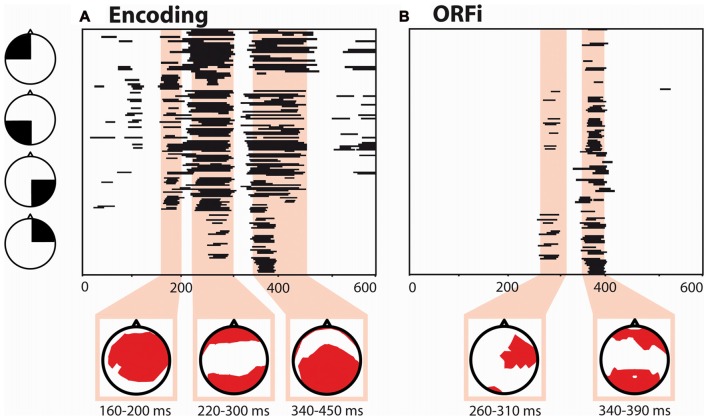
Global waveform analysis. Electrode- and time-wise global waveform analysis for the Encoding condition and ORFi condition displayed according to scalp position (black triangles). **(A)** Main effects in the one-way repeated measure ANOVA (rmANOVA) with factor Stimulus (New1, OneBack1, N-Back1). **(B)** Interactions in the the 2 × 2 rmANOVA with factor Stimulus (New, Ten) and Run (1,2). Black lines indicate significant effects (*p* < 0.01 for ≥20 ms). Electrodes with significant effects are displayed in red.

The time- and electrode-wise ANOVA for Encoding (OneBack1 in comparison with New1 stimuli and N-Back1) revealed a statistically significant (*p* < 0.01; ≥20 ms) main effect of Stimulus at 160–200 ms, 220–300 ms and 340–450 ms (Figure 2A). For the ORFi condition (comparison of New2 in comparison with New1, N-Back1 and N-Back2), there was a statistically significant (*p* < 0.01; ≥20 ms) Stimulus × Run interaction at 260–310 ms and later at 340–390 ms (Figure 2B).

### Frontal Waveform Analysis

**Figure 3:**
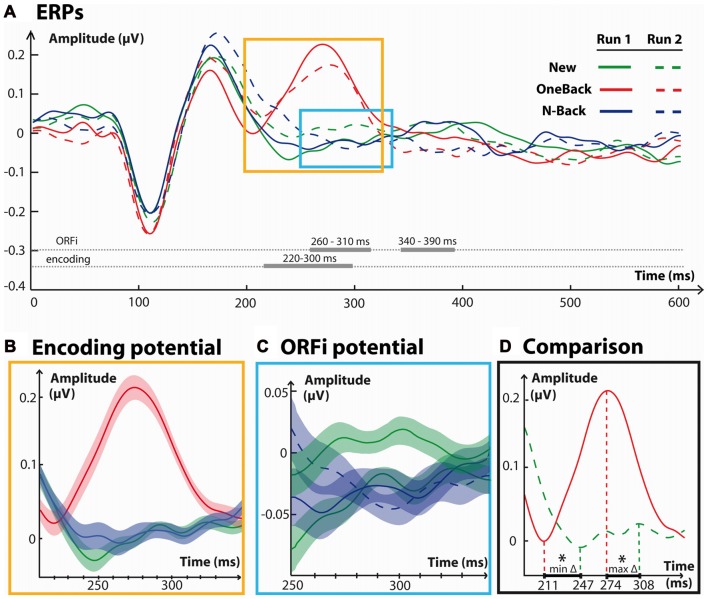
Frontal waveform analysis. **(A)** Waveforms from a cluster of eight neighboring frontal electrodes averaged over 600 ms for each condition. Traces in a straight line refer to stimuli from run 1, while traces in a dashed line refer to run 2. Horizontally, in thick gray lines are indicated the time windows with significant effects between stimuli of each condition. Time windows with effects specific to the Encoding (220–300 ms) and ORFi (260–310 ms) potentials are detailed in the colored boxes. **(B)** Event related potentials (ERPs) in response to New1, OneBack1 and N-Back1 stimuli. **(C)** ERPs in response to New1, New2, N-Back1 and N-Back2 stimuli **(C)** for the period of significant interaction between their respective amplitudes. **(D)** Comparison of the positive potentials in ERPs from OneBack1 and New2 stimuli for the period of time identified in **(B,C)**. The red dashed lines indicate the time points of average minimum and maximum amplitude. The lagging time period between minima and maxima was statistically significant, as indicated asterisks (*), and is projected in bold on the *x* axis. The Encoding potential precedes ORFi potential by approximately 35 ms.

Figure 3A summarizes the results obtained over the frontal electrode group. As is evident from this figure, main effects appeared around the time window of 200–300 ms. ANOVAs were performed in each of the time-windows reported in the global waveform analysis to assess the direction of effects.

#### Encoding

OneBack1 induced a strong potential at about 220–300 ms which was significantly more positive than New1 and N-Back1 (Main effect of Stimulus, *F*_(2,34)_ = 8.3, *p* < 0.01, $\eta_{\text{p}}^{\text{2}}$ = 0.67; Figure 3B). There were no statistical effects for this comparison in the frontal cluster in the 160–200 ms time-window (*p* = 0.051) or in the 340–450 ms time-window (*p* = 0.08). Figure 3A shows that a very similar frontal potential with the same latency was evoked by OneBack2 items, that is, immediate repetitions within the second run.

#### ORFi

Over the 260–310 ms time period there was a significant Stimulus × Run interaction (*F*_(1,17)_ = 4.46, *p* = 0.04, $\eta_{\text{p}}^{\text{2}}$ = 0.21). New2 stimuli triggered a significantly more positive potential than New1, N-Back1 and N-Back2 stimuli (*p* < 0.05; Figure 3C). Over this period there was also a main effect of Run (*F*_(1,17)_ = 7.28, *p* = 0.02, $\eta_{\text{p}}^{\text{2}}$ = 0.30), but not Stimulus (*p* = 0.3). Over the 340–390 ms period, there was a significant Stimulus × Run interaction (*F*_(1,17)_ = 26.3, *p* < 0.01, $\eta_{\text{p}}^{\text{2}}$ = 0.61). N-Back2 were significantly more positive than New2 and N-Back1 (*p* < 0.05) but not New1. There were no main effects of Run (*p* = 0.14) nor Stimulus (*p* = 0.19).

The potential representative of Encoding (OneBack1) significantly differed from compared stimuli at 220–330 ms; the potential specific for ORFi (New2) at 260–310 ms.

#### Comparison of Timing

A central question of the present study concerned the precise timing between ORFi and Encoding. Figure 3D shows the direct comparison between the two stimuli of main interest, OneBack1 (Encoding) and New2 (ORFi). The potential in response to OneBack1 had a significantly earlier onset (211 ± 16 ms), i.e., time point of the early minimum amplitude, than the potential in response to New2 (247 ± 38 ms; *t*_(17)_ = 4.3, *p* < 0.01). Concordantly, the OneBack1 potential reached its peak amplitude significantly earlier (274 ± 27 ms) than New2 (308 ± 41 ms; *t*_(17)_ = 2.9, *p* < 0.01). The end of the potentials can only be estimated, because the amplitude of the OneBack1 potential continued to decrease for a prolonged period. Visual inspection (Figure 3D) suggests that both responses terminated around 330 ms (OneBack2 had a more conspicuous end at 330 ms). Thus, both the beginning and the peak of the potential in response to OneBack1 (Encoding) preceded the response to New2 (ORFi) by about 35 ms but both ended at about the same time.

### Source Analysis

**Figure 4:**
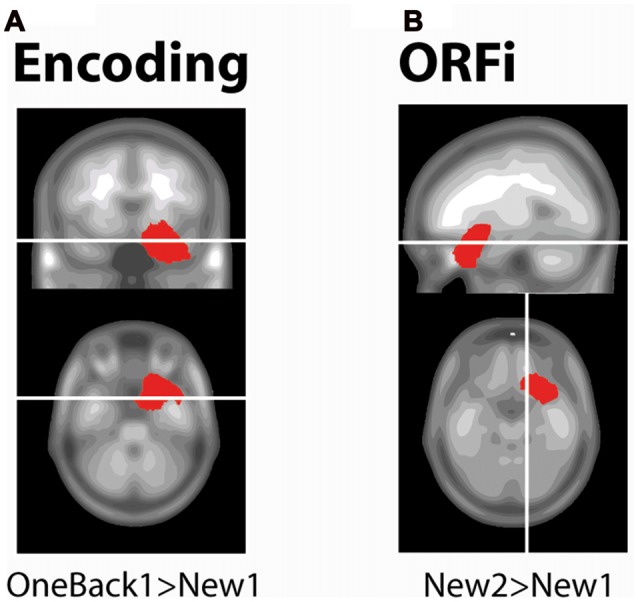
Source estimations. Estimated current densities differences between stimuli as determined with paired *t*-tests. White lines indicate the slices selected. Red areas depict solution points with statistically significant differences (*p* < 0.01 for ≥20 ms). **(A)** The encoding condition is exampled with statistical comparison of New1 and OneBack1. There is a stronger activation in the medial temporal lobe (MTL) in response to OneBack1 than New1 at 274 ms, which corresponds to the time of maximum ERP amplitude for OneBack1.** (B)** The ORFi condition is exampled with statistical comparison of New2 and New1. There is a stronger activation in the orbitofrontal cortex (OFC) in response to New2 than New1 at 308 ms, which corresponds to the time of maximum ERP amplitude for New2.

This analysis served to determine the brain areas active during Encoding (220–300 ms) and ORFi (260–310 ms; >20 ms, *p* < 0.01). At the time point of peak amplitude (274 ms), OneBack1 stimuli induced stronger current density than New1 in right MTL (Figure 4A, *t*_(17)_ > 7.56, *p* < 0.01). At the time point of its peak amplitude (308 ms), New2 induced stronger current density than New1 in the right orbitofrontal area (Figure 4B, *t*_(17)_ > 2.95, *p* < 0.01).

### Functional Connectivity

**Figure 5:**
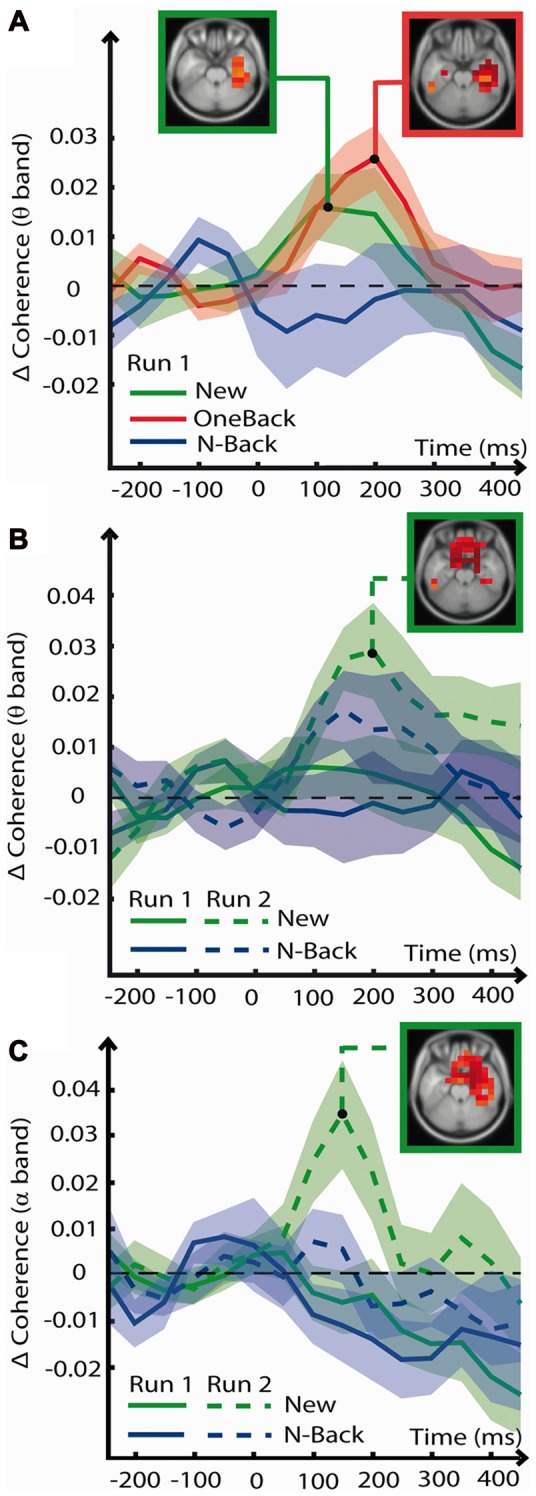
Coherence analysis. Event-Related Coherence (ERCoh) between voxels of various brain areas in various frequencies of oscillation as a function of time (*x* axis) and coherence change from baseline (*y* axis). When relevant for a specific stimulus, voxels of significant ERCoh change (*t*_(17)_ > 2.57, *p* < 0.01, cluster correction) are displayed in a box on a slice of MRI template. **(A)** ERCoh in the theta frequency from voxels of the right hippocampal complex (HC) in response to stimuli of the encoding condition (New1, Oneback1 and N-Back1). Voxels with significant ERCoh increase are displayed in the red box for OneBack1 in the MTL at 200 ms and in the green box for New1 also in the MTL at 100 ms. **(B)** ERCoh in the theta frequency from voxels of the OFC in response to stimuli of the ORFi condition (New1, New2, N-Back1 and N-Back2). Voxels with significant ERCoh change in the OFC at 200 ms and in response to New2 are displayed in the green box. **(C)** ERCoh in the alpha frequency from voxels of the OFC in response to the ORFi condition. Voxels with significant ERCoh change at 150 ms in response to New2 are visible in the OFC and MTL and are displayed in the green box.

This analysis served to explore global modulations of connectivity specific for Encoding and ORFi. Figure 5 summarizes the result. Results discussed in this section were statistically significant and survived cluster correction as described in the methods (*t*_(17)_ > 2.57, *p* < 0.01).

#### Encoding

Multidimensional matrices in each frequency band revealed various fluctuations of ERCoh. In the theta band frequency, OneBack1 stimuli induced a significant increase of coherence over baseline between 100 and 300 ms (peak at about 200 ms) centered on the right MTL (Figure 5A, red). The OneBack1 ERCoh increase was preceded (peak at 100 ms) by a smaller increase of coherence (Figure 5A, green) in response to New1, also localized in the MTL. N-Back1 stimuli did not induce an increase of coherence at any time point (*p* > 0.05). Pair-wise comparisons confirmed that there was a greater increase of ERCoh for OneBack1 than for N-Back1 but not than for New1 (*p* > 0.05).

In the alpha band frequency there was a general decrease of ERCoh after 200 ms in the right MTL, which was unspecific for any stimulus. In the beta and gamma frequencies there were no fluctuations of coherence.

#### ORFi

Multidimensional matrices revealed fluctuations of ERCoh in the alpha and theta band frequencies. In response to New2 stimuli, theta coherence increased above baseline between 100 and 300 ms in the OFC (Figure 5B). Pair-wise comparisons confirmed that ERCoh was greater during processing of New2 than New1 and N-Back1, but not than N-Back2 (*p* > 0.05).

In the alpha band frequency there was an increase of coherence from baseline in response to New2 at 150–200 ms, localized in the OFC and right MTL (Figure 5C). Pair-wise comparisons confirmed that ERCoh was greater in both OFC and right MTL during processing of New2 compared to New1, N-Back1, and N-Back2 ERCoh. There were no fluctuations of coherence in the beta and gamma frequencies.

### Seed Analysis

To further explore the coherence between brain regions identified with global FC analysis, that is, the OFC and right MTL, we performed a seed analysis of these regions.

#### Right Medial Temporal Lobe

**Figure 6:**
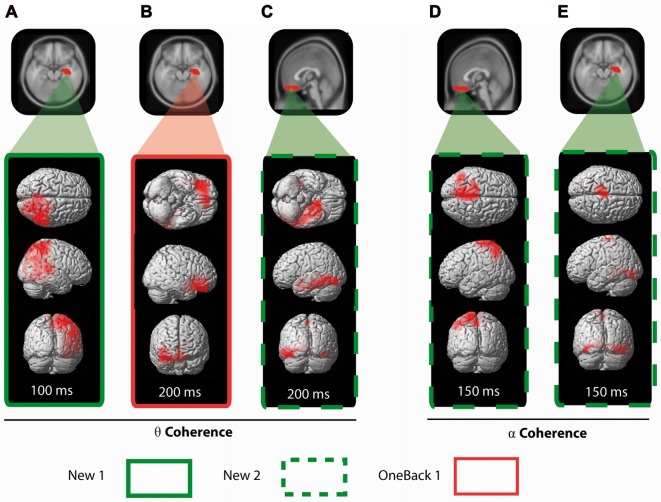
Seeded coherence analysis. For each stimulus previously identified with a significant ERCoh increase, the average ERCoh of the same frequency was seeded in a selected brain area (in red on the MRI template) and compared to every other voxel of the brain. The results are displayed in a 3D representation of the functionally synchronized areas for each stimulus. In the theta band frequency: **(A)** New1 is seeded with the right HC and results are displayed a 100 ms. **(B)** OneBack1 is also seeded with the rightHC and results are displayed at 200 ms. **(C)** New2 is seeded with OFC and results are displayed at 200 ms. In the alpha band frequency: New2 is seeded with the OFC **(D)** and with the right HC **(E)** and results are displayed at 150 ms.

We defined a seed in the right hippocampal complex (HC) as part of the MTL and calculated the average ERCoh of voxels from this area to each other voxel (Figure 6). The main finding was that, at 200 ms, OneBack1 induced a significant increase of ERCoh above baseline in theta band between the right HC (MTL) seed region and the OFC (Figure 6B). A secondary finding was that, at 100 ms already, ERCoh in response to New1 increased above baseline in the theta band frequency between the right HC seed and the right inferior parietal area (Figure 6A, red).

Incidentally, New2 (measure of ORFi) also induced a strong increase of coherence in the alpha band at 200 ms, namely, between the right HC seed and the parietal region (Figure 6E).

#### Orbitofrontal Cortex

We defined a seed region in the gyrus rectus as part of the OFC (Figure 6C). New2 provoked a strong increase of coherence between the OFC and the left MTL at 200 ms in the theta band range (Figure 6C). In the same period (around 200 ms), New2 induced a strong increase of coherence in the alpha band between the OFC and the parietal region at 200 ms (Figure 6D).

In summary, the right HC had increased coherence in the theta band with the OFC in response to OneBack1 items and with right parietal area in response to New1 items. First presentation of items in the second run (New2) triggered an increase of theta coherence between the gyrus rectus and the left MTL. Finally, New2 items triggered an increase of alpha coherence of both the OFC and right HC with the parietal cortex.

## Discussion

This study indicates that as thoughts undergo ORFi (i.e., the verification of whether thoughts pertain to ongoing reality or not), they are simultaneously encoded about 200–300 ms after their evocation. Encoding, as measured with our task, starts slightly before ORFi. The study demonstrates a complex, frequency-specific interaction in this period between the OFC and the MTL and of these regions with the (parietal) neocortex.

The electrophysiological markers used to examine ORFi and encoding had known behavioral correlates. The present study, similar to previous ones (Schnider et al., 2000, 2002; Treyer et al., 2003, 2006; Wahlen et al., 2011; Bouzerda-Wahlen et al., 2015), demonstrated a frontal positivity (or rather: attenuation of a negativity) at about 200–300 ms during the successful processing of the stimuli (New2) associated with OFC activation. Failure in processing this kind of stimuli has been a reliable marker of reality confusion in patients with damage to the OFC or directly connected structures, whereas non-confabulating subjects normally processed these stimuli (Schnider et al., 1996b; Schnider and Ptak, 1999; Nahum et al., 2012).

Immediate picture repetition evoked a very strong, conspicuous positive frontal potential also at about 200–300 ms, similar to a previous study (James et al., 2009). Inverse solutions used in the present study again point to the MTL as the generator of this signal. While the hippocampal provenance of this potential has been confirmed by depth electrode recordings in two patients evaluated for epilepsy surgery (Nahum et al., 2011), its role for encoding is less straightforward. According to task requirements, it might reflect recollection rather than encoding. However, there is concordance among many studies that recollection of previously encountered material induces prolonged potential modulations starting at the earliest at about 350 ms, mostly over more posterior electrodes (Ranganath and Paller, 1999; Rugg and Curran, 2007; Addante et al., 2012). This is considerably later than the potential observed in the present study. The Encoding potential is also reminiscent of the P300, a potential associated with attentional processes predictive of memory encoding (Polich, 2007). However, the P300 is typically pronounced over more posterior electrodes, in contrast to the frontal emphasis of the Encoding signal in our study, and it is typically elicited by stimuli popping out from a series of repeated stimuli (Polich, 2007), which is partly opposite to the potential induced by immediate stimulus repetition and presented in our study. Our earlier observation that increased theta-coherence of the MTL in the period of this potential is associated with better recognition after 30 min would still be compatible with an attentional effect indirectly supporting encoding of the stimuli (Thézé et al., 2016), as postulated for the P300 (Polich, 2007). However, this interpretation is hardly consistent with the fact that the degree of amnesia, as observed in patients with Wernicke-Korsakoff syndrome, was inversely related to the strength of this potential (Nahum et al., 2015). These observations make it likely that the frontal potential in response to immediately repeated stimuli indeed reflects MTL-conveyed encoding, or at least a process supporting encoding. While a more obvious encoding task might be desirable for future studies, this potential is probably the best-substantiated marker of MTL-conveyed encoding to date.

Following this logic, the present study provides a precise timing between the two processes of interest. The ORFi potential had an onset at ~245 ms, peaked at ~310 ms, lasted for about 80 ms and ended around 330 ms. The Encoding potential started and peaked about 35 ms earlier than the ORFi potential: it started at ~210 ms after stimulus presentation, peaked at ~275 ms and lasted until 330 ms, with a total duration of about 120 ms. For the sake of this study, the two processes had to be studied in a dissociated fashion by their respective markers. In a natural setting, however, these processes would be expected to exert a concerted action on an upcoming memory trace. This study suggests that, during the whole period in which the trace undergoes reality filtering, it is also being encoded.

The coherence analysis performed here hints at a specific interaction between OFC and MTL in this period. The initial coherence analysis including the whole brain, with no preselected regions, detected a circumscribed increase of theta coherence in the OFC with the rest of the brain at about 200–300 ms in response to New2 stimuli, that is, during ORFi. A seed analysis indicated that the main partner area in this coherence was the MTL. Conversely, in the same period around 200 ms, stimuli representative of Encoding (OneBack items) provoked increased theta coherence specifically in the MTL. Seed analysis centered on the HC indicated that the coherence increase mainly targeted the OFC.

Increased theta coherence in the human MTL has previously been associated with encoding and with maintenance in working memory (Rutishauser et al., 2010; Battaglia et al., 2011; Fell and Axmacher, 2011). Both interpretations are compatible with the postulated significance of the immediately repeated items in the present study. Synchronization in other frequencies (gamma, alpha/beta) recorded with depth electrodes has also been associated with memory encoding (Fell et al., 2008; Fell and Axmacher, 2011). In the present study, both the OFC and the MTL had increased coherence in the alpha band with the parietal cortex, possibly influencing the neocortical representation of the memory trace.

The increased theta coherence observed here is likely subserved by direct connections between the OFC and MTL (Barbas and Blatt, 1995; Carmichael and Price, 1995; Cavada et al., 2000). Interaction between these structures, as seen with functional MRI (Ranganath et al., 2005; Zeithamova et al., 2012) or as reflected in increased theta coherence in humans (Battaglia et al., 2011; Fell and Axmacher, 2011), has previously been linked to successful memory formation (Nieuwenhuis and Takashima, 2011). Increased OFC-MTL coherence in the theta band, as observed here, has also been reported when rats had to choose the arm of a Y-maze that would currently lead to reward (Benchenane et al., 2010) or when they had to choose between two stimuli having opposing reward associations in two spatial contexts (Place et al., 2016). While these experiments were interpreted with reference to reward processing, they closely reflect the challenges on which reality-confusing patients fail: to sense which thought (memory) applies to the present moment (Nahum et al., 2009, 2012; Schnider et al., 2017).

The concerted action of ORFi and encoding is thought to leave a memory trace, which, when evoked tomorrow, will be easily attributed to a real event or an imagination. That is, encoding during ORFi today allows for reality monitoring tomorrow. Reality and source monitoring have been suggested to rely on richer spatial and temporal contextual attributes and semantic details (Johnson and Raye, 1981; Johnson et al., 1993; Mitchell and Johnson, 2009), thus, memory characteristics established at encoding. Concordantly, functional MRI studies demonstrated that stimulus processing at encoding is a critical variable for later reality monitoring, although areas of brain activity were extremely heterogeneous (Davachi et al., 2003; Gonsalves et al., 2004; Kensinger and Schacter, 2005; Sugimori et al., 2014). Laboratory test of reality and source monitoring typically demand an effortful search in memory and induce evoked potential responses from 400 ms on Leynes et al. (2005), Hayama et al. (2008), Rosburg et al. (2011) and Bouzerda-Wahlen et al. (2015). This slow response may reflect the extended reasoning processes postulated to underlie source memory decisions (Johnson and Raye, 1981; Johnson et al., 1993; Mitchell and Johnson, 2009). While this postulate may hold for delicate distinctions of highly similar memories, such as tested in typical laboratory tasks, it appears little plausible for everyday reality monitoring, which is commonly effortless and immediate.

Our study has limitations. First, the marker of Encoding (immediately repeated stimuli), inducing a brief evoked potential at 200–300 ms, does not represent all there is to memory encoding. Constitution of source information necessary for reality monitoring may depend on the long process of memory consolidation, which was not targeted by our study. Second, while clinical studies clearly indicate that deficient ORFi prevents subsequent reality monitoring (Schnider et al., 2005; Schnider, 2008), our present study did not directly explore this link. All our study provides is a plausible timing and direction of MTL-OFC interactions. Notwithstanding these caveats, we suggest that MTL-conveyed encoding during OFC-mediated reality filtering provides an efficient explanation for most instances of reality monitoring in everyday life.

## Author Contributions

RT: conception of the research, design of protocol, data collection, data analysis, and writing of the article. ALM: data collection, data analysis, writing of the article and revision of the article. LN: conception of the research, design of protocol, data collection and revision of the article. AGG: data analysis, writing of the article and revision of the article. AS: funding, conception of the research, design of protocol, writing of the article and revisions.

## Conflict of Interest Statement

The authors declare that the research was conducted in the absence of any commercial or financial relationships that could be construed as a potential conflict of interest.

## Footnotes

^1^http://www.fbmlab.com/cartool-software/

^2^http://www.unil.ch/line/en/home/menuinst/about-the-line/software--analysis-tools.html

^3^http://www.nitrc.org/plugins/mwiki/index.php/nutmeg:MainPage

## Abbreviations

ERCoh: Event Related Coherence
ERP: Event Related Potential
HC: Hippocampus
MTL: Medio-Temporal Lobe
OFC: Orbitofrontal Cortex
ORFi: Orbitofrontal Reality Filtering
rmANOVA: repeated measure ANOVA.
